# HRR as a predictor of lung health: insights from the NHANES database

**DOI:** 10.3389/fmed.2025.1503142

**Published:** 2025-02-24

**Authors:** Jiaji Zhou, Wenyi Du, Hanzhou Huang, Yongqi Chen, Huixing Li, Leyan Chen, Feng Liu, Mingfeng Zheng

**Affiliations:** ^1^Department of Thoracic Surgery, Wuxi People's Hospital, Nanjing Medical University, Wuxi Medical Center, Wuxi, China; ^2^Wuxi People's Hospital Affiliated with Nanjing Medical University, Wuxi, China; ^3^Department of General Surgery, The Affiliated Wuxi People's Hospital of Nanjing Medical University, Wuxi, China

**Keywords:** chronic respiratory disease, HRR, pulmonary function, NHANES, cross-sectional study

## Abstract

**Background:**

Chronic respiratory diseases (CRPD) are a global health threat characterized by oxidative stress, systemic inflammation, hypoxemia, and respiratory distress. Inflammatory indicators such as hemoglobin-to-red blood cell distribution width ratio (HRR) have been explored in relation to diseases of the respiratory system, but the correlation between HRR and pulmonary function has not been established. As part of this study, a representative sample of the National Health and Nutrition Examination Survey (NHANES) respondents aged 40 or over was used to examine the correlation between HRR and pulmonary function indices.

**Methods:**

Data from the 2007–2012 NHANES were used for this study. HRR and four pulmonary function parameters were compared using regression and subgroup analyses. The Restricted Cubic Spline (RCS) model was employed to find out if there are any non-linear relationships between these associations. Multiple sensitivity analyses were used to verify the correlation between the two.

**Results:**

After adjusting for confounding variables, the data showed that for each unit increase in HRR among the population as a whole, for each unit increase in HRR, FVC increased by 0.11, FEV1 increased by 0.22, peak expiratory flow (PEF) increased by 0.24 and forced expiratory flow at 25–75% (FEF25-75%) was elevated by 0.49. In addition, we determined linear and positive correlations between FVC, FEV1, PEF or PEF 25–75% and HRR by constructing the RCS model curves. The positive correlation between HRR and pulmonary function parameters was affirmed through sensitivity analysis. Furthermore, except for the PEF 25–75%, FVC, FEV1, PEF all showed a significant upward trend with the increase of HRR in non-Hispanic white female population.

**Conclusion:**

According to our study, HRR was positively correlated with FVC, FEV1, PEF, and PEF25-75% in a middle-aged and older adult US population. It would be useful to study the specific impact of HRR on pulmonary function and to investigate the potential pathophysiological mechanisms that might link them.

## 1 Introduction

The primary classifications of CRPD encompass chronic obstructive pulmonary disease (COPD) and asthma (1). The condition is primarily characterized by airflow limitation (AL). The incidence of CRPD is high worldwide and is increasing year by year (1, 2). CRPD prevalence and severity are silent plagues that silently erode society’s health, as reported in the 2019 Global Burden of Disease (GBD) study (3). The global prevalence of CRPD exceeded 400 million, and the number of deaths exceeded 4 million, an increase of 28.5 and 39.8%, respectively (3). Among them, deaths from COPD exceed 3 million, the leading cause of death from CRPD. In addition, there are more than 200 million cases of asthma, which has the highest prevalence among CRPD (3). All of this indicates the current grim situation of CRPD. Therefore, screening for CRPD at an early stage, before it becomes symptomatic and progresses, is crucial.

At an early stage of CRPD, lung function assessment has advantages (4–6). However, lung function is only tested at the onset of respiratory symptoms. Furthermore, lung function testing is not widely used as part of health screening or primary care (7, 8). According to a US study, since the implementation of the National Lung Health Education Program (NLHEP), although the use of spirometers in primary care to detect and manage COPD has improved, 70% of patients diagnosed with COPD still do not undergo spirometry (9). In an epidemiological context, a simple biological marker that can be used to screen for lung dysfunction in the early stages of the disease could be helpful in its assessment, management, and treatment.

Red cell distribution width (RDW) is an advantageous hematological indicator for detecting heterogeneity in red blood cell volume. It is commonly used in clinical practice to diagnose and differentiate anaemia (10). RDW levels, are dynamic indicators of the body’s homeostasis, responding to internal and external stimuli. Clinical surveys have demonstrated that RDW has been linked to various diseases, and its impact on mortality is independent of other clinical factors (11–13). Hemoglobin (HGB), the oxygen-carrying protein found in large quantities in red blood cell (RBC) (14, 15), is presently the most widely utilized biochemical marker for the diagnosis of anemia in blood (16, 17). The ratio of HRR is calculated by dividing HGB by RDW. This is a recently proposed inflammatory marker by Sun et al. (18). There is a strong correlation between low HRR levels and poor prognosis, shorter disease-free survival, and disease progression among other adverse outcomes (19–22). The association between HRR and pulmonary function is potentially attributable to the interplay of various biological mechanisms, including oxygen transport and supply–demand equilibrium, inflammatory response, neuroendocrine regulation, and oxidative stress. Insufficient oxygen intake alters erythrocyte production, impacting HGB and increasing RDW due to reduced deformability and more immature erythrocytes. Inflammatory cytokines lower HGB, damage vascular endothelium, and activate the sympathetic nervous system and RAAS, affecting RDW and HGB. Additionally, free radicals damage erythrocytes, influencing HRR. These mechanisms influence alterations in HRR by modulating erythrocyte production and function, as well as blood components, thereby serving as indicators of pulmonary function status.

Unlike spirometry, HRR and RDW can indicate early lung disease changes, which may miss early damage. Blood tests for HRR and RDW are more convenient and repeatable, requiring only blood samples and allowing for dynamic disease monitoring over time. While HRR and RDW exhibit certain attributes, such as potential early alterations in pulmonary diseases, ease of detection, and repeatability, they are not exclusive to pulmonary conditions. Multiple factors influence these measures, provide an indirect reflection of impacts on the hematological system, and possess limitations in diagnostic precision and the assessment of pulmonary function impairment. In U.S. middle-aged and older adult populations, no correlation between HRR and pulmonary function has been explored as of yet. This survey uses NHANES data to explore the relationship between HRR and pulmonary function in this demographic and to assess HRR as a potential early indicator of lung function issues.

## 2 Methods

### 2.1 Data source

**Figure 1:**
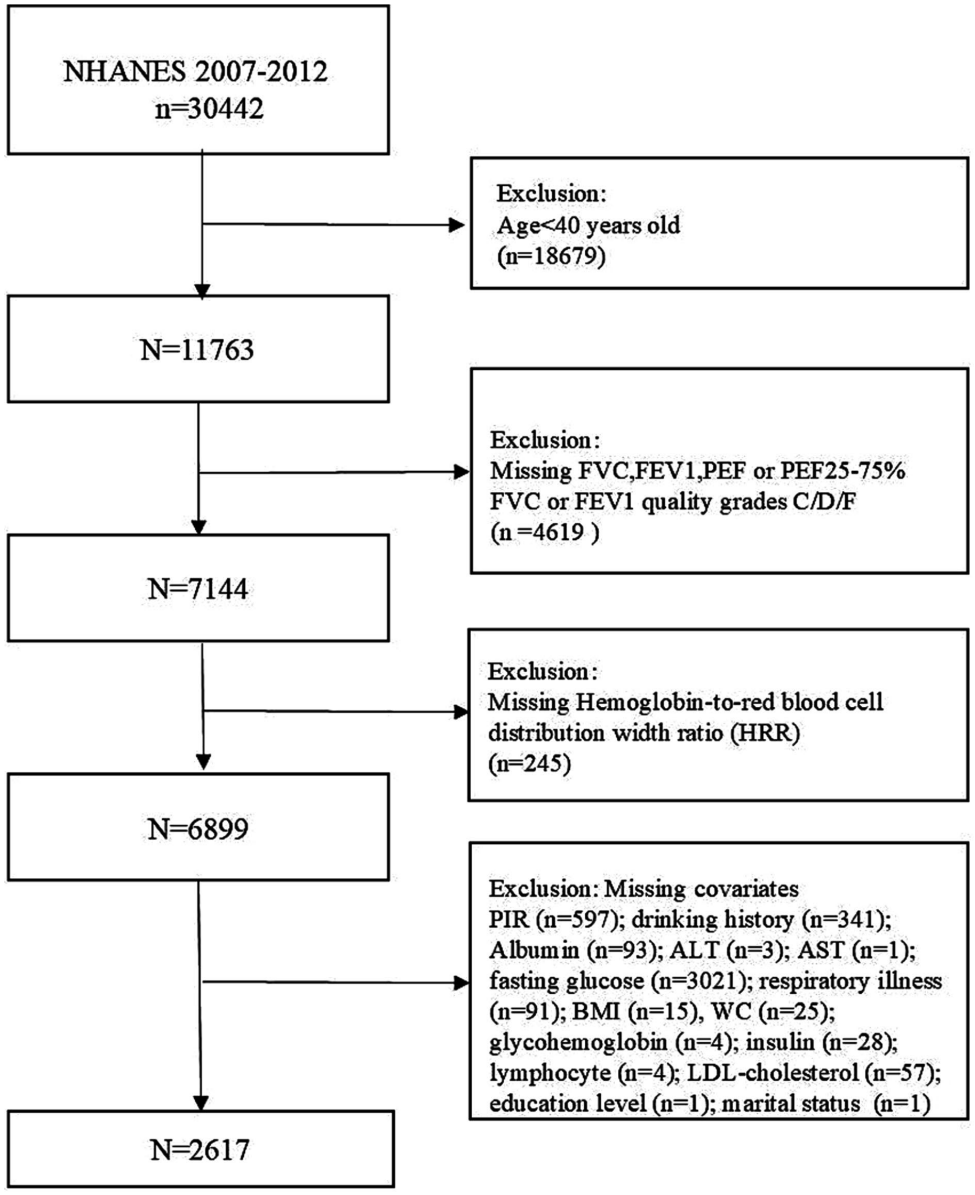
Flowchart of participant selection.

The present survey employed data from the NHANES between 2007 and 2012. To guarantee the comprehensiveness and precision of the findings, stringent inclusion and exclusion criteria were applied. Individuals below the age of 40 (*n* = 18,679) were excluded, as were those with missing pulmonary function test outcome data (FVC, FEV1, PEF or PEF25-75%) or low data quality (C, D, F) (*n* = 4,619). Similarly, participants who were involved in the study of incomplete HRR data (*n* = 245) and those with other missing data (*n* = 4,282) on at least one of the following covariates were also excluded: The variables included in the analysis were poverty-to-income ratio (PIR), drink history, alanine aminotransferase (ALT); aspartate aminotransferase (AST), creatinine, uric acid, glycohemoglobin, monocyte number, HGB, waist circumference (WC), body mass index (BMI), smoking history and so on. In conclusion, this study encompasses a substantial, accurate representation of the United States population. For purposes of clarity, Figure 1 presents a flow chart delineating the screening process. All participants provided informed written consent before participating in the NHANES study, and the National Center for Health Statistics verified all data prior to public release.

### 2.2 Lung function assessment

Lung function was evaluated utilizing the Ohio 822/827 dry-roll volume spirometer in accordance with the guidelines established by the American Thoracic Society (ATS) and the European Respiratory Society (ERS). The primary spirometric variables measured included FVC, FEV1, PEF, and PEF 25–75%. It is essential to ensure that the results of the measurement are reliable and accurate, the ATS/ERS criteria for acceptability and reproducibility were rigorously applied, resulting in quality grades ranging from A to F, with grades A and B denoting acceptable measurements.

### 2.3 HRR measurement

For each participant, HRR was computed by dividing HGB (measured in grams per deciliter, g/dL) by RDW, with results rounded to two decimal places. Information on blood indicators is derived from a representative sample of NHANES data, including such as CRP (mg/dL), WBC (1,000 cells/uL), HRR, RDW (%), and HGB (g/dL). Detailed protocols for sample collection and laboratory testing are documented in the Laboratory/Medical Technician Procedures Manual, which can be accessed from the NHANES website.

### 2.4 Other covariates

In order to guarantee the thoroughness of the study, a number of additional variables were included, based on the findings of previous research and the collective experience of the clinical team. A demographic dataset provided by the NHANES contains variables such as sex, age, race (other Hispanic, non-Hispanic white, Mexican American, others, non-Hispanic black), educational level, and poverty-income ratio (PIR). Smoking history (over the course of their lifetime, they have smoked at least 100 cigarettes), drinking history (consumed a minimum of 12 alcoholic beverages per year.), BMI, high blood pressure, and respiratory illness were derived from questionnaire data available on the NHANES website. The level of education was divided into three categories: high school or GED, above high school, and less than high school. Weight can be classified into three categories based on BMI: obesity (>30.0 kg/m^2^), overweight (25.0–30.0 kg/m^2^), and below average or healthy weight (>25.0 kg/m^2^). Individuals of married or cohabiting status are classified into the first category; divorced, separated, or widowed individuals are classified as the second category; and those who are unmarried are classified as reconstituted. Visit the NHANES website for more information on these covariates.

### 2.5 Statistical analysis

Continuous variables obtained from participants were assessed for normality based on data characteristics. Variables that conformed to normal distribution were expressed as mean ± standard deviation (SD), and those that did not conform to normal distribution were expressed as median and interquartile range (IQR) using non-parametric tests. Simultaneously, frequencies and percentages are employed to characterize categorical variables. Furthermore, the analysis of categorical variables is conducted utilizing the chi-square test or Fisher’s exact test when the expected frequency is less than five. Continuous variables were tested using linear regression, and the Wilcoxon rank-sum test was employed to assess the differences between the groups. Lung function parameters were ln transformed for normal distribution before modeling. Subsequently, four models of linear regressions were employed to examine the correlation between HRR and pulmonary function indicators. Crude Model was unadjusted, excluding any covariates. Model 2 incorporated key demographic variables, including sex, age, and race. Model 3 extended Model 2 by adding BMI. Model 4 adds education level, marital status, poverty-to-income ratio (PIR), drinking history, smoking history, alanine aminotransferase (ALT), aspartate aminotransferase (AST), creatinine, uric acid, glycohemoglobin, monocyte number, hemoglobin (HGB), and waist circumference on the basis of Model 3 (23). The fully adjusted models were also used to explore possible stratified associations between HRR and pulmonary function. In addition, RCS smoothed curve fitting was used to determine whether there is a relationship between the two variables. Sensitivity analyses, such as standardization processes, reverse causation analysis, new models, and studies on specific populations, were employed to further demonstrate the correlation between HRR and pulmonary function parameters.

These statistical methods enabled comprehensive analyses examining possible HRR and lung function associations. Statistical analyses were conducted utilizing R (version 4.2.0) and MSTATA (version 0.92). Statistical significance is typically determined by 0.05 or less as a *p*-value.

## 3 Results

### 3.1 Baseline characteristics of the participants

Among the participants from the 2007–2012 NHANES survey, Table 1 provides demographic, exam, laboratory, and questionnaire information. A total of 2,617 participants, 1,294 males, and 1,323 females, were incorporated into our research. Participants in the study were 56 years old on average, with non-Hispanic whites comprising the majority of the study population at 51.4%. 75% consumed a minimum of twelve alcoholic beverages annually, and 50.2% smoked over 100 cigarettes in their lifetime. Further details are shown in Table 1.

**Table 1:** Baseline characteristics of the selected participants.

Characteristic	Sex			*p*-value
	Overall, *N* = 2,617	Female, *N* = 1,323	Male, *N* = 1,294	
**Age**	56 (47, 65)	56 (47, 65)	56 (48, 65)	0.680
Race/ethnicity				0.772
Mexican American	364 (13.9%)	180 (13.6%)	184 (14.2%)	
Non-Hispanic Black	488 (18.6%)	254 (19.2%)	234 (18.1%)	
Non-Hispanic White	1,346 (51.4%)	687 (51.9%)	659 (50.9%)	
Other Hispanic	270 (10.3%)	132 (10.0%)	138 (10.7%)	
Other Race	149 (5.7%)	70 (5.3%)	79 (6.1%)	
Education Level				0.113
Above high school	1,414 (54.0%)	739 (55.9%)	675 (52.2%)	
High school or GED	588 (22.5%)	293 (22.1%)	295 (22.8%)	
Less than high school	615 (23.5%)	291 (22.0%)	324 (25.0%)	
Marital status				<0.001
Divorced, separated, or widowed	692 (26.4%)	435 (32.9%)	257 (19.9%)	
Married or cohabiting	1,708 (65.3%)	770 (58.2%)	938 (72.5%)	
Unmarried	217 (8.3%)	118 (8.9%)	99 (7.7%)	
PIR				0.070
Low [0,1.65]	866 (33.1%)	462 (34.9%)	404 (31.2%)	
Medium [1.65,4.04]	877 (33.5%)	443 (33.5%)	434 (33.5%)	
High [4.04,5]	874 (33.4%)	418 (31.6%)	456 (35.2%)	
Body mass index (kg/m^**2**^**)**				<0.001
< 25	640 (24.5%)	356 (26.9%)	284 (21.9%)	
≥ 30	1,033 (39.5%)	574 (43.4%)	459 (35.5%)	
25–30	944 (36.1%)	393 (29.7%)	551 (42.6%)	
HRR				<0.001
Low [0.26,1.06]	870 (33.2%)	645 (48.8%)	225 (17.4%)	
Medium [1.06,1.18]	873 (33.4%)	500 (37.8%)	373 (28.8%)	
High [1.18,1.58]	874 (33.4%)	178 (13.5%)	696 (53.8%)	
Smoking history				<0.001
No	1,304 (49.8%)	758 (57.3%)	546 (42.2%)	
Yes	1,313 (50.2%)	565 (42.7%)	748 (57.8%)	
High blood pressure				0.761
No	1,470 (56.2%)	747 (56.5%)	723 (55.9%)	
Yes	1,147 (43.8%)	576 (43.5%)	571 (44.1%)	
Drinking history				<0.001
No	653 (25.0%)	469 (35.4%)	184 (14.2%)	
Yes	1,964 (75.0%)	854 (64.6%)	1,110 (85.8%)	
Respiratory illness				0.361
No	2,113 (80.7%)	1,059 (80.0%)	1,054 (81.5%)	
Yes	504 (19.3%)	264 (20.0%)	240 (18.5%)	
**Glycohemoglobin (%)**	5.60 (5.30, 6.00)	5.60 (5.30, 6.00)	5.60 (5.30, 6.00)	0.324
**Albumin (g/dL)**	4.20 (4.00, 4.40)	4.20 (4.00, 4.30)	4.30 (4.10, 4.40)	<0.001
**ALT (U/L)**	22 (17, 29)	20 (16, 25)	25 (20, 33)	<0.001
**AST (U/L)**	24 (20, 28)	22 (19, 27)	25 (22, 30)	<0.001
**Creatinine (μmol/L)**	74 (64, 88)	66 (58, 75)	84 (74, 95)	<0.001
**Uric acid (mg/dL)**	5.50 (4.60, 6.40)	4.90 (4.10, 5.80)	6.00 (5.30, 6.80)	<0.001
**Fasting Glucose (mg/dL)**	102 (95, 113)	100 (93, 109)	104 (97, 116)	<0.001
**Insulin (uU/mL)**	10 (7, 16)	10 (6, 16)	11 (7, 17)	0.101
**Lymphocyte number (1,000 cells/uL)**	1.90 (1.50, 2.30)	1.90 (1.60, 2.30)	1.80 (1.50, 2.20)	<0.001
**Monocyte number (1,000 cells/uL)**	0.50 (0.40, 0.60)	0.50 (0.40, 0.60)	0.50 (0.40, 0.60)	<0.001
**White blood cell count (1,000 cells/uL)**	6.30 (5.30, 7.60)	6.20 (5.10, 7.60)	6.30 (5.30, 7.60)	0.124
**HGB (g/dL)**	14.30 (13.30, 15.20)	13.60 (12.70, 14.30)	15.10 (14.30, 15.90)	<0.001
**Red cell distribution width (%)**	12.70 (12.30, 13.30)	12.70 (12.30, 13.50)	12.70 (12.30, 13.30)	0.011
**Platelet count (1,000 cells/uL)**	238 (201, 283)	254 (216, 299)	224 (190, 259)	<0.001
**LDL-cholesterol (mg/dL)**	118 (96, 142)	120 (99, 144)	116 (94, 140)	0.008
**Waist Circumference (cm)**	100 (91, 110)	97 (88, 108)	102 (94, 112)	<0.001
**FVC**	8.19 (7.98, 8.39)	8.02 (7.86, 8.17)	8.38 (8.23, 8.50)	<0.001
**FEV1**	7.91 (7.70, 8.11)	7.75 (7.59, 7.91)	8.09 (7.92, 8.24)	<0.001
**PEF**	8.93 (8.74, 9.13)	8.79 (8.62, 8.92)	9.12 (8.96, 9.25)	<0.001
**PEF 25–75%**	7.75 (7.38, 8.06)	7.64 (7.30, 7.92)	7.89 (7.49, 8.19)	<0.001

### 3.2 The associations between HRR and lung function parameters

In accordance with Table 2, multiple regression analyses were used to test the association between HRR and lung function parameters. In Crude Model and Model 2, HRR demonstrated a positive association with FVC, FEV1, PEF, and the PEF 25–75%. Crude Model illustrates the unadjusted association, whereas Model 2 accounts for adjustments based on sex, age, and racial factors. In Model 3, adjustments were made for BMI based on the parameters established in Model 2, similar results were observed. Finally, in analyses that are fully adjusted population-wide, HRR was positive for FVC [*β* (95% CI) = 0.11 (0.01, 0.21)], FEV1 [β (95% CI) = 0.22 (0.10, 0.33)], PEF [β (95% CI) = 0.24 (0.11, 0.36)] and PEF 25–75% [β (95% CI) = 0.49 (0.23, 0.74)].

**Table 2:** Multivariate regression model analysis among HRR and lung function paraments.

**Characteristic**	Model 1		Model 2		Model 3		Model 4	
	β (95% CI)	*p* value	β (95% CI)	*p* value	β (95% CI)	*p* value	β (95% CI)	*p* value
FVC	0.73 (0.67, 0.79)	< 0.001	0.61 (0.55, 0.67)	< 0.001	0.58 (0.52, 0.64)	< 0.001	0.11 (0.01, 0.21)	0.028
FEV1	0.70 (0.64, 0.77)	< 0.001	0.14 (0.08, 0.20)	< 0.001	0.13 (0.07, 0.19)	< 0.001	0.22 (0.10, 0.33)	< 0.001
PEF	0.63 (0.56, 0.69)	< 0.001	0.14 (0.08, 0.21)	< 0.001	0.15 (0.08, 0.21)	< 0.001	0.24 (0.11, 0.36)	< 0.001
PEF 25–75%	0.63 (0.50, 0.75)	< 0.001	0.19 (0.06, 0.32)	0.003	0.23 (0.10, 0.36)	< 0.001	0.49 (0.23, 0.74)	< 0.001

Crude Model: no covariates were adjusted. Model 2: sex, age, and race/ethnicity were adjusted. Model 3: Model 2 plus BMI was adjusted. Model 4: Model 3 plus education level, marital status, PIR, drink history, smoking history, ALT, AST, creatinine, uric acid, glycohemoglobin, monocyte number, HGB, and waist circumference. HRR, hemoglobin-to-red blood cell distribution width ratio, CI, confidence interval; PIR, poverty-income ratio; BMI, body mass index. ALT, alanine aminotransferase; AST, aspartate aminotransferase.

### 3.3 Assessment of the association between HRR and pulmonary function parameters on a stratified basis

**Figure 2:**
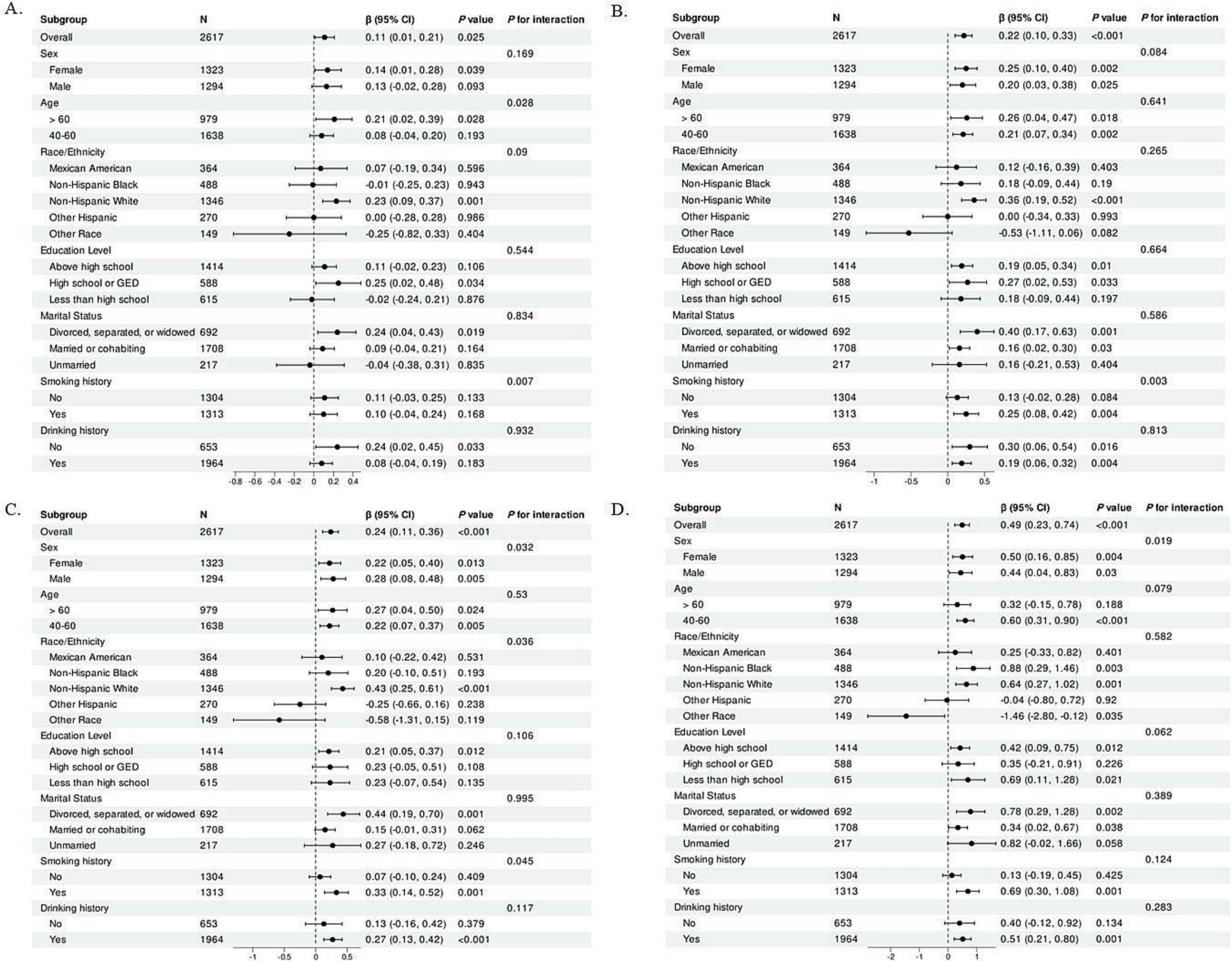
Subgroup analysis of the association of HRR with **(A)** FVC, **(B)** FEV1, **(C)** PEF, and **(D)** PEF 25–75%. The above was adjusted for sex, age, race/ethnicity, BMI, education level, marital status, PIR, drink history, smoking history, ALT, AST, creatinine, uric acid, glycohemoglobin, monocyte number, HGB, and waist circumference. In each case, the model was not adjusted for the stratification variable itself. HRR, hemoglobin-to-red blood cell distribution width ratio, CI, confidence interval; PIR, poverty-income ratio; BMI, body mass index; ALT, alanine aminotransferase; AST, aspartate aminotransferase.

Stratified data analysis was conducted to determine whether the results from the multivariate regression analysis examined the relationship between HRR and functional indices of lung parameters across various subgroups were stable. Figure 2 depicts the results.

In the subgroup analyses, FEV1 generally rises as HRR increases among individuals of non-Hispanic white and those with a high school education or higher. Similarly, FVC demonstrated an upward trend with the augmentation of HRR in most subgroups such as females, aged over 60 years, non-Hispanic white, high school or GED education, divorced, separated, or widowed, and less than 100 cigarettes smoked in a lifetime. In contrast, HRR was positively associated with PEF in the subgroups non-Hispanic whites, above high school education, divorced, separated, or widowed, drinkers of 12 or more drinks per year, and lifetime smokers of 100 or more cigarettes; similar results were observed for HRR and PEF 25–75% in the same subgroups. Except for PEF 25–75%, there is an interaction between smoking history and other pulmonary function parameters. There was also an interaction between age and FVC and an interaction between sex and PEF 25–75%. Furthermore, an interaction also exists among sex, race, and PEF.

### 3.4 Linear relationship between HRR and lung function parameters

**Figure 3:**
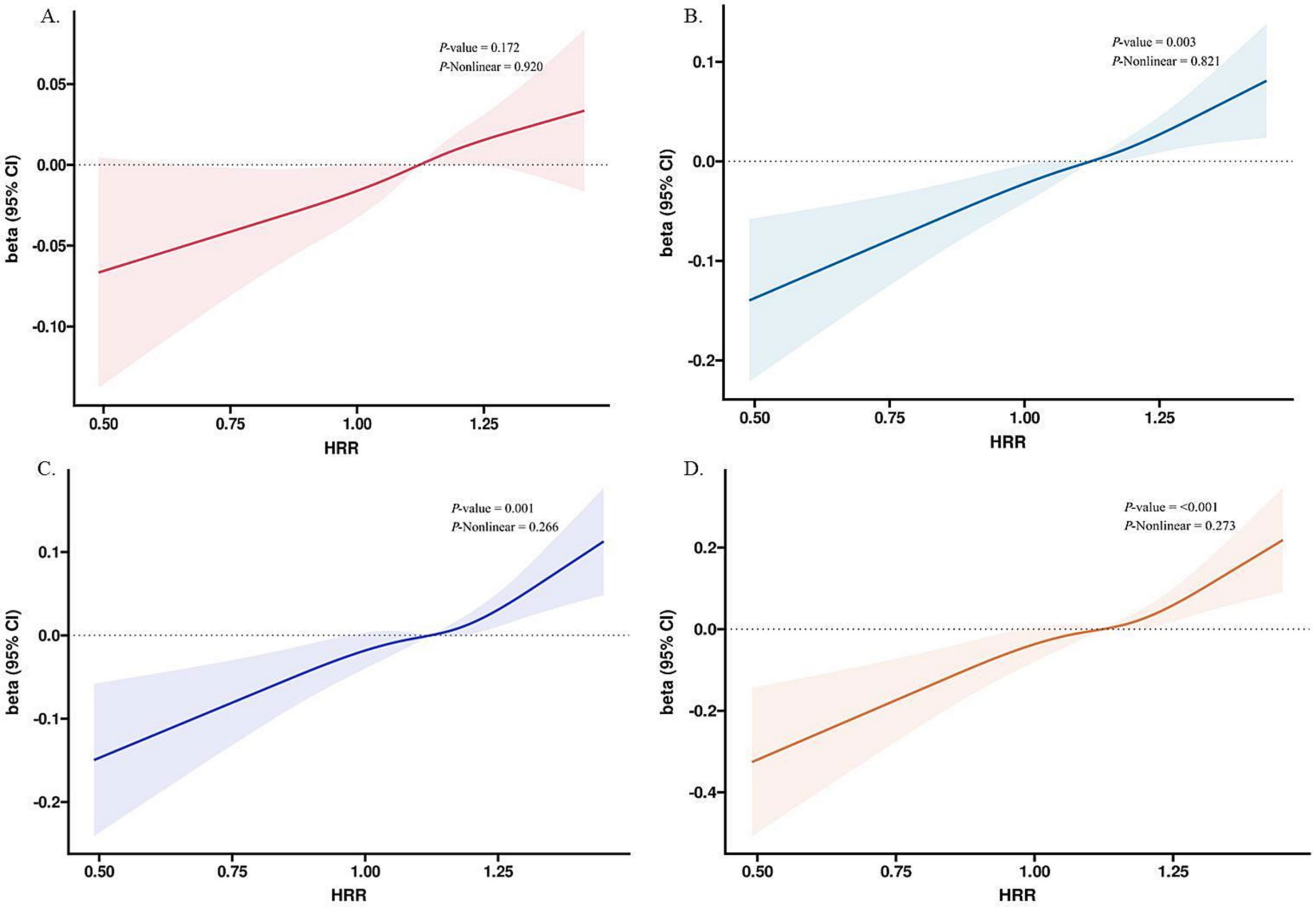
Restricted cubic spline analyses the association of HRR with **(A)** FVC, **(B)** FEV1, **(C)** PEF, and **(D)** PEF 25–75%. Adjusted for sex, age, race/ethnicity, BMI, education level, marital status, PIR, drink history, smoking history, ALT, AST, creatinine, uric acid, glycohemoglobin, monocyte number, HGB, and waist circumference. HRR, hemoglobin-to-red blood cell distribution width ratio, CI, confidence interval; PIR, poverty-income ratio; BMI, body mass index; ALT, alanine aminotransferase; AST, aspartate aminotransferase.

A linear relationship has been empirically validated between HRR and FVC (nonlinear *p* = 0.920) (Figure 3A), FEV1 (nonlinear *p* = 0.821) (Figure 3B), PEF (nonlinear *p* = 0.266) (Figure 3C), and PEF 25–75% (nonlinear *p* = 0.277) (Figure 3D) by RCS model and smoothing the curve fitting. FVC, FEV1, PEF, and PEF 25–75% showed a significant increasing trend with increasing HRR. Results are shown in Figure 3.

### 3.5 Sensitivity analysis

Multivariate regression analysis after the standardization of HRR verified the correlation between HRR and pulmonary function parameters (Supplementary Table 1). Moreover, the reverse causal relationship between the two was also demonstrated (Supplementary Table 2). In accordance with Supplementary Table 3, multiple regression analyses were used to test the association between HRR and lung function parameters. In Models 5 and 6, HRR demonstrated a positive association with FVC, FEV1, PEF, and the PEF 25–75%. In Model 5, adjustments were made for high blood pressure based on the parameters established in Model 4, similar results were observed. Finally, in analyses that are fully adjusted population-wide, HRR was positive for FVC [*β* (95% CI) = 0.11 (0.01, 0.21)], FEV1 [β (95% CI) = 0.21 (0.10, 0.32)], PEF [β (95% CI) = 0.23 (0.10, 0.36)] and PEF 25–75% [β (95% CI) = 0.46 (0.21, 0.72)]. Furthermore, among non-Hispanic white women, except for the PEF 25–75%, FVC, FEV1, and PEF all showed a significant upward trend with the increase of HRR. Results are shown in Supplementary Table 4.

## 4 Discussion

HRR and lung function parameters are only briefly studied in the United States in people who are middle-aged and older. Researchers examined 2,617 older and middle-aged adults between 2007 and 2012 to determine whether HRR was related to lung function parameters. An analysis of the relationship between HRR and four pulmonary function parameters was conducted using four linear regression models with multiple coefficients. According to the 2007–2012 NHANES data, Model 2 was adjusted to account for variations in basic covariate, and unadjusted Crude Model showed that a positive correlation was found between HRR and FVC, FEV1, PEF, and PEF 25–75%. BMI was taken into account in Model 3, and HRR was correlated with the above-mentioned pulmonary function parameters. Lastly, Model 4 has been thoroughly refined, and analyzed in the overall population, HRR was associated with FVC [*β* (95% CI) = 0.11 (0.01, 0.21)], FEV1 [β (95% CI) = 0.22 (0.10, 0.33)], PEF [β (95% CI) = 0.24 (0.11, 0.36)], and PEF 25–75% [β (95% CI) = 0.49 (0.23, 0.74)]. To assess the precision and robustness of this correlation, a stratified analysis was performed, and further exploratory subgroup analyses showed that with the exception of PEF 25–75%, interactions were observed between smoking history and various pulmonary function parameters. Additionally, interactions were identified between age and FVC, as well as between sex and PEF 25–75%. Moreover, a further interaction was noted among sex, race, and PEF. Given the inherent differences in baseline characteristics, hormonal levels, and other factors between male and female pulmonary functions (24), these variations may account for the observed significant disparities in the impact of HRR on pulmonary function parameters across sex subgroups. The substantial variation in the influence of HRR on pulmonary function parameters across subgroups with differing smoking histories may be attributed to the fact that smoking elevates the risk of respiratory tract inflammation and airway obstruction (25). Individuals from diverse racial backgrounds possess distinct genetic profiles that may influence lung development and physiological functions. Concurrently, environmental and lifestyle factors, such as levels of air pollution, dietary practices, and exercise habits, also differ across racial groups, potentially exerting indirect effects on pulmonary function. In addition, socioeconomic variables, which are often closely intertwined with race, can impact access to and utilization of healthcare resources, thereby influencing the monitoring of lung function and the efficacy of disease treatment. These multifaceted differences may contribute to the observed disparities in the effects of HRR on pulmonary function parameters among racial subgroups (26–30). Furthermore, prolonged smoking can result in severe pulmonary conditions, such as COPD. The reliability of the results was further assessed by constructing an RCS model for curve fitting.

This study’s results show direct correlations, suggesting that an increase in HRR may cause an increase in FVC, FEV1, PEF, and PEF 25–75%. Although there are relatively few existing findings on the relationship between HRR and lung function, the use of HRR as a novel inflammatory marker to predict the prognosis of lung-related diseases has become a current research hotspot. Chen JL et al. evaluate the value of RDW, neutrophil-to-lymphocyte ratio (NLR), and HRR in the progression of non-small cell lung cancer (NSCLC), drawing the conclusion: RDW, NLR, and HRR have demonstrated significant discriminatory power between NSCLC patients and healthy controls. Furthermore, RDW and NLR exhibit a positive correlation with the stage of NSCLC, whereas HRR shows a negative correlation with the disease stage (31). In response to this conclusion, subsequent ROC curve analysis demonstrated that the combined use of RDW, NLR, HRR, and CEA exhibits substantial diagnostic efficacy, with an AUC of 0.925 (95% CI: 0.897–0.954), sensitivity of 79.60%, and specificity of 93.60%. This combination can serve as a straightforward and effective biomarker for the diagnosis and progression assessment of NSCLC. A single-center retrospective cohort study found that low HRR was associated with overall survival (OS) in patients with pulmonary large-cell neuroendocrine carcinoma (PLCNEC) (22). Wu F et al. conducted a study to evaluate the prognostic value of HRR in small cell lung cancer (SCLC) and showed that low HRR was associated with OS [HR (95% CI) = 3.782 (2.151–6.652)] and progression-free survival (PFS) [HR (95% CI) = 2.112 (1.195–3.733)] and was an independent predictor of poor prognosis (32). These findings align with the outcomes of our research, which showed that HRR was positively correlated with lung function parameters. However, Liu S et al. showed that HRR exhibited a negative correlation exclusively with overall mortality rates among patients diagnosed with COPD, and there was no significant correlation with mortality associated with chronic lower respiratory disease (CLRD) (33). In the Liu-S study, COPD was defined using NHANES questionnaire data, where participants were considered to have COPD if they answered ‘yes’ to any of these questions: “Have you ever been diagnosed by a physician with chronic bronchitis, emphysema, or COPD?.” Participants under 18 and pregnant women were excluded (33). However, the lung function parameters in our study were derived from NHANES examination data, which excluded participants under 40 years of age. The heterogeneity among the reported studies—encompassing variations in comorbidity data, statistical analyses, demographic data, etc—renders it challenging to ascertain the association between HRR and lung function. This variability may account for the discrepancies observed in the reported epidemiological studies.

However, no studies have investigated its relationship with pulmonary function parameters. Therefore, our goal is to investigate the possible relationship between HRR and pulmonary function. To the extent of our current understanding, this survey constitutes the inaugural cross-sectional study investigating the relationship between HRR and lung function. The research encompassed a sample size of 2,617 cases, and the results included four lung function-related indicators: FVC, FEV1, PEF, and PEF25-75%. A thorough stratified analysis encompassing examination, personal history, comorbidity data, and demographics was conducted to validate the precision and robustness of the findings. Accordingly, our study showed that corrected HRR was positively correlated with FVC, FEV1, PEF, and PEF25-75% in the general US middle-aged and older adult population.

Currently, the specific mechanism linking HRR and lung function is not clear. To the best of our knowledge, the main possible mechanisms are as follows: First, inflammation plays a central role in COPD’s pathophysiology (34). Findings have substantiated that RDW and HRR are associated with inflammatory processes, thereby establishing them as novel biomarkers indicative of inflammation (10, 35–37). Studies have shown that high levels of RDW are associated with oxidative stress and inflammatory states (10, 38, 39). Oxidative stress markedly influences erythrocyte homeostasis and viability, contributing to an elevated RDW through the acceleration of erythrocyte turnover (40). Inflammation may lead to an increase in RDW by inhibiting erythropoiesis and disrupting the clearance of immature erythrocytes (41). In addition, low hemoglobin levels are a key indicator of the inflammatory process. Research has substantiated a correlation between hemoglobin levels and the prognosis of patients with COPD, indicating that anemia is linked to a poorer prognosis in this patient population (42, 43). The HRR, as determined by this specific calculation method, may serve as an indirect indicator of pulmonary oxygen supply and the oxygen-carrying capacity of the blood. A diminished HRR value could suggest abnormalities within the hematological system, potentially impairing normal pulmonary function. For instance, a reduction in HGB levels or an increase in RDW resulting in decreased HRR might indicate conditions such as anemia or abnormal erythrocyte maturation, which could adversely affect pulmonary oxygen uptake and transport. Consequently, monitoring HRR variations may facilitate the timely detection of hematological issues that could compromise lung health, thereby offering valuable insights for early intervention. In chronic pulmonary disorders such as COPD, persistent inflammation and hypoxia can influence hematological parameters. HRR may exhibit heightened sensitivity to these alterations, potentially manifesting abnormalities during the early stages of the disease or upon changes in the patient’s condition. For instance, as COPD advances, exacerbated anemia or alterations in erythrocyte morphology may arise, consequently affecting HRR. In patients with established pulmonary diseases, HRR may serve as a valuable biomarker for monitoring disease progression and evaluating therapeutic efficacy. An improvement in HRR during treatment may suggest that the therapeutic interventions have effectively enhanced blood supply and pulmonary function. In conclusion, HRR, a composite metric derived from RDW, serves as an informative parameter for elucidating the relationship between these hematological indices and COPD.

This study has multiple advantages. In this study, we used a nationally representative cohort of middle-aged and older adult individuals in the United States to assess the correlation between HRR and lung function, which means that the data represents a general sample of the American population with high-quality and standardized measurements. In addition, this study represents the inaugural investigation into the relationship between HRR and pulmonary function. Secondly, we accounted for potential confounders by adjusting for covariates such as socio-demographic factors, lifestyle variables, and BMI. Thirdly, subgroup analyses were conducted to elucidate the differential impact of HRR on lung function between various subpopulations. Lastly, using fully adjusted models, RCS curve fitting was employed to further explore the relationship between HRR and pulmonary function parameters.

The principal limitations of our study are its cross-sectional design, which precludes the establishment of causal and temporal relationships between HRR and lung function. Additionally, a prolonged follow-up period may be necessary to thoroughly evaluate the relationship between HRR and lung function. Our sample size was insufficient to exclude individuals with specific diseases that could impact lung function, thereby limiting our ability to account for numerous factors that may influence lung function. Future research should consider investigating a larger population. Finally, the relationship between HRR and lung function remains to be fully elucidated.

## 5 Conclusion

Our findings demonstrate that, within a fully adjusted model, HRR is positively associated with FVC, FEV1, PEF, and PEF25-75% among middle-aged and older adult populations in the United States. These results underscore the significance of HRR as a potential determinant of lung function in this demographic.

## Data Availability

The datasets presented in this study can be found in online repositories. The names of the repository/repositories and accession number(s) can be found at: https://www.cdc.gov/nchs/nhanes/index.htm.
