# Supplementary material for: HRR as a predictor of lung health: insights from the NHANES database
Source: Front Med (Lausanne). 2025 Feb 24;12:1503142. doi: 10.3389/fmed.2025.1503142 (PMC11891021; doi:10.3389/fmed.2025.1503142)
Supplement: Supplementary file 1 [file Table_1.docx]

**Supplementary Table 1: Multivariate regression model analysis among Standardized HRR and lung function paraments**

| **Characteristic** | **Crude Model** | |  | **Model 2** | |  | | **Model 3** | |  | | **Model 4** | |
| --- | --- | --- | --- | --- | --- | --- | --- | --- | --- | --- | --- | --- | --- |
|  | **β (95% CI)** | ***P* value** |  | **β (95% CI)** | ***P* value** |  | **β (95% CI)** | | ***P* value** |  | **β (95% CI)** | | ***P* value** |
| FVC | 0.12 (0.11, 0.13) | < 0.001 |  | 0.02 (0.01, 0.03) | < 0.001 |  | 0.02(0.01, 0.02) | | < 0.001 |  | 0.02 (0.01, 0.03) | | 0.028 |
| FEV1 | 0.11 (0.10, 0.12) | < 0.001 |  | 0.02 (0.01, 0.03) | < 0.001 |  | 0.02 (0.01, 0.03) | | < 0.001 |  | 0.02 (0.02, 0.05) | | < 0.001 |
| PEF | 0.10 (0.09, 0.11) | < 0.001 |  | 0.02 (0.01, 0.03) | < 0.001 |  | 0.02 (0.01, 0.03) | | < 0.001 |  | 0.04 (0.02, 0.06) | | < 0.001 |
| PEF 25-75% | 0.10 (0.08, 0.12) | < 0.001 |  | 0.03 (0.01, 0.05) | 0.003 |  | 0.04 (0.02, 0.06) | | < 0.001 |  | 0.08 (0.04, 0.12) | | < 0.001 |

Crude Model: no covariates were adjusted

Model 2: sex, age, and race/ethnicity were adjusted

Model 3: Model 2 plus BMI was adjusted.

Model 4: Model 3 plus education level, marital status, PIR, drink history, smoking history, ALT, AST, creatinine, uric acid, glycohemoglobin, monocyte number, HGB, and waist circumference.

HRR, hemoglobin-to-red blood cell distribution width ratio, CI, confidence interval; PIR, poverty-income ratio; BMI, body mass index;

ALT, alanine aminotransferase; AST, aspartate aminotransferase.
