# Supplementary material for: HRR as a predictor of lung health: insights from the NHANES database
Source: Front Med (Lausanne). 2025 Feb 24;12:1503142. doi: 10.3389/fmed.2025.1503142 (PMC11891021; doi:10.3389/fmed.2025.1503142)
Supplement: Supplementary file 3 [file Table_3.docx]

**Supplementary Table 3: Multivariate regression model analysis among HRR and lung function paraments**

| **Characteristic** | **Model 5** | |  | **Model 6** | |
| --- | --- | --- | --- | --- | --- |
|  | **β (95% CI)** | ***P* value** |  | **β (95% CI)** | ***P* value** |
| FVC | 0.12 (0.02, 0.22) | 0.020 |  | 0.11 (0.01, 0.21) | 0.029 |
| FEV1 | 0.23 (0.11, 0.34) | < 0.001 |  | 0.21 (0.01, 0.32) | < 0.001 |
| PEF | 0.25 (0.12, 0.37) | < 0.001 |  | 0.23 (0.10, 0.36) | < 0.001 |
| PEF 25-75% | 0.50 (0.25, 0.76) | < 0.001 |  | 0.46(0.21, 0.72) | < 0.001 |

Model 5: sex, age, race/ethnicity, BMI, education level, marital status, PIR, drink history, smoking history, ALT, AST, creatinine, uric acid, glycohemoglobin, monocyte number, HGB, waist circumference and high blood pressure were adjusted

Model 6: Model 5 plus albumin was adjusted.

HRR, hemoglobin-to-red blood cell distribution width ratio, CI, confidence interval; PIR, poverty-income ratio; BMI, body mass index;

ALT, alanine aminotransferase; AST, aspartate aminotransferase.
