# Supplementary material for: HRR as a predictor of lung health: insights from the NHANES database
Source: Front Med (Lausanne). 2025 Feb 24;12:1503142. doi: 10.3389/fmed.2025.1503142 (PMC11891021; doi:10.3389/fmed.2025.1503142)
Supplement: Supplementary file 4 [file Table_4.docx]

**Supplementary Table 4: Multivariate regression model analysis among HRR and lung function paraments in** **Non-Hispanic white female population**

| **Characteristic** | **Crude Model** | |  | **Model 2** | |  | | **Model 3** | |  | | **Model 4** | |
| --- | --- | --- | --- | --- | --- | --- | --- | --- | --- | --- | --- | --- | --- |
|  | **β (95% CI)** | ***P* value** |  | **β (95% CI)** | ***P* value** |  | **β (95% CI)** | | ***P* value** |  | **β (95% CI)** | | ***P* value** |
| FVC | 0.21 (0.09, 0.33) | < 0.001 |  | 0.19 (0.09, 0.29) | < 0.001 |  | 0.14(0.04, 0.24) | | 0.005 |  | 0.37 (0.18, 0.57) | | < 0.001 |
| FEV1 | 0.20 (0.05, 0.34) | 0.008 |  | 0.17 (0.05, 0.29) | 0.006 |  | 0.15 (0.03, 0.27) | | 0.017 |  | 0.52 (0.29, 0.75) | | < 0.001 |
| PEF | 0.17 (0.04, 0.31) | 0.013 |  | 0.16 (0.03, 0.28) | 0.016 |  | 0.16 (0.03, 0.29) | | 0.014 |  | 0.55 (0.31, 0.80) | | < 0.001 |
| PEF 25-75% | 0.10 (-0.20, 0.40) | 0.520 |  | 0.05 (-0.22, 0.32) | 0.704 |  | 0.12 (-0.12, 0.39) | | 0.393 |  | 0.85 (0.33, 1.37) | | 0.001 |

Crude Model: no covariates were adjusted

Model 2: age was adjusted

Model 3: Model 2 plus BMI was adjusted.

Model 4: Model 3 plus education level, marital status, PIR, drink history, smoking history, ALT, AST, creatinine, uric acid, glycohemoglobin, monocyte number, HGB, and waist circumference.

HRR, hemoglobin-to-red blood cell distribution width ratio, CI, confidence interval; PIR, poverty-income ratio; BMI, body mass index;

ALT, alanine aminotransferase; AST, aspartate aminotransferase.
